# Supplementary material for: Zinc Oxide-Loaded Cellulose-Based Carbon Gas Sensor for Selective Detection of Ammonia
Source: Nanomaterials (Basel). 2023 Dec 15;13(24):3151. doi: 10.3390/nano13243151 (PMC10745631; doi:10.3390/nano13243151)
Supplement: Supplementary file 1 [file nanomaterials-13-03151-s001.zip › nanomaterials-2742470-supplementary.pdf]

Supplementary Materials

Zinc Oxide-Loaded Cellulose-Based Carbon Gas Sensor for Selective Detection of Ammonia

Hao Xu, Zhu-Xiang Gong, Li-Zhu Huo, Chao-Fei Guo, Xue-Juan Yang, Yu-Xuan Wang * and Xi-Ping Luo *

Zhejiang Provincial Key Laboratory of Chemical Utilization of Forestry Biomass, College of Chemistry and Materials Engineering, Zhejiang A&F University, Hangzhou 311300, China; jasonxu@stu.zafu.edu.cn (H.X.); 2022005@stu.zafu.edu.cn (Z.-X.G.); hlz@stu.zafu.edu.cn (L.-Z.H.); chaoguo@zafu.edu.cn (C.-F.G.); yangxj@zafu.edu.cn (X.-J.Y.)

* Correspondence: 20190050@zafu.edu.cn (Y.-X.W.); luoxiping@zafu.edu.cn (X.-P.L.); Tel.: +86-131-2238-0892 (Y.-X.W.); +86-159-6888-2838 (X.-P.L.)

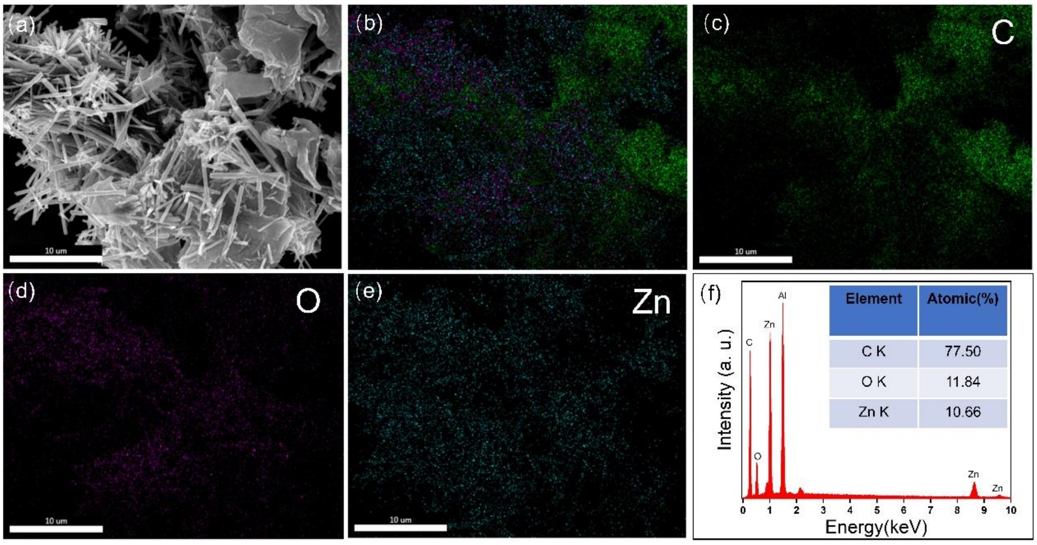

Figure S1. (a-e) SEM images of the ZnO/CBC-60% and corresponding mapping images of C, O and Zn; (f) the EDS analysis of the prepared ZnO/CBC-60%.

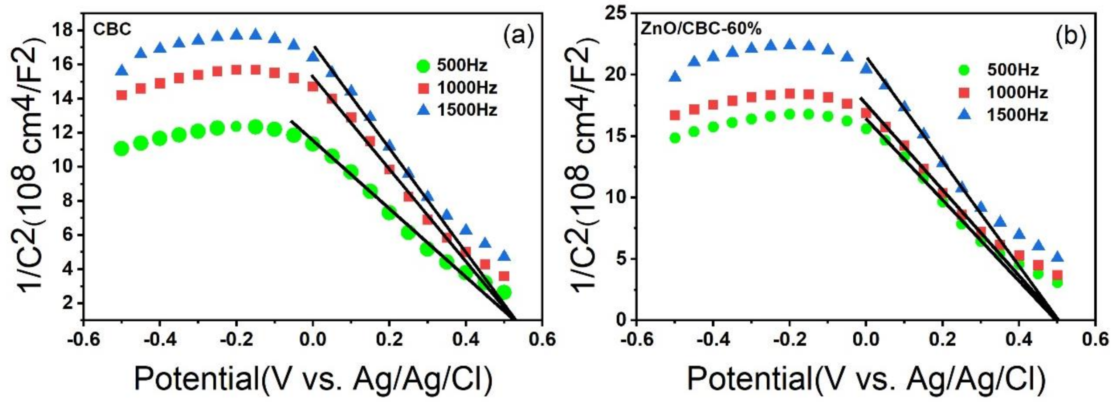

Figure S2. Mott-Schottky curves of (a) CBC, (b) ZnO/CBC-60%
